# Molecular Epidemiology and Genotyping of *Mycobacterium tuberculosis* Isolated in Baghdad

**DOI:** 10.1155/2014/580981

**Published:** 2014-02-26

**Authors:** Ruqaya Mustafa Ali, Alberto Trovato, David Couvin, Amina N. Al-Thwani, Emanuele Borroni, Fahim H. Dhaer, Nalin Rastogi, Daniela M. Cirillo

**Affiliations:** ^1^Genetic Engineering and Biotechnology Institute for Post Graduate Studies, University of Baghdad, 10070 Jadriyah, Baghdad, Iraq; ^2^Emerging Bacterial Pathogens Unit, San Raffaele Scientific Institute (HSR), via Olgettina, 60 20132 Milano, Italy; ^3^Institut Pasteur de la Guadeloupe, Abymes, 97183 Guadeloupe, France; ^4^Ministry of Agriculture, Al Nidhal Street, Baghdad 5157, Iraq

## Abstract

Tuberculosis (TB) remains a major health problem in Iraq but the strains responsible for the epidemic have been poorly characterized. Our aim was to characterize the TB strains circulating in Bagdad (Iraq). A total of 270 *Mycobacterium tuberculosis* complex (MTBC) strains isolated between 2010 and 2011 from TB patients attending the Center of Chest and Respiratory diseases in Baghdad were analyzed by Spoligotyping. The analysis indicated that 94.1% of the isolates belong to known genotype clades: CAS 39.6%, ill-defined T clade 29.6%, Manu 7.4%, Haarlem 7%, Ural 4.1%, LAM 3.3%, X 0.7%, LAM7-TUR 0.7%, EAI 0.7%, S 0.7%, and unknown 5.9%. Comparison with the international multimarker database SITVIT2 showed that SIT 309 (CAS1-Delhi) and SIT1144 (T1) were the most common types. In addition, 44 strains were included in SITVIT2 database under 16 new Spoligotype International Types (SITs); of these, 6 SITs (SIT3346, SIT3497, SIT3708, SIT3790, SIT3791, and SIT3800) (*n* = 32 strains) were created within the present study and 10 were created after a match with an orphan in the database. By using 24-loci MIRU-VNTR-typing on a subset of 110 samples we found a high recent transmission index (RTI) of 33.6%. In conclusion, we present the first unifying framework for both epidemiology and evolutionary analysis of *M. tuberculosis* in Iraq.

## 1. Introduction

Tuberculosis (TB) is an ancient disease that currently represents an immense global health challenge. In 2011, WHO estimated that globally there were 8.7 million new cases of active TB leading to 1.4 million reported deaths [1].

According to the 2012 report of the Ministry of Health (MOH), the incidence rate of TB in Iraq was 45/100,000, with 13,860 new TB cases and 1140 of previously treated cases. The Iraqi laboratory network includes 124 district smear microscopy laboratories and one national reference laboratory located in Baghdad performing cultures and drug susceptibility testing of *M. tuberculosis* [2].

In the last decades, a large number of different molecular methods based on DNA fingerprints have been developed. The usefulness of these methods has been demonstrated primarily as epidemiological markers to discriminate the pathogen at the genus, species, and subspecies level. The level of strain differentiation is of crucial importance for the study of transmission dynamics, determining whether the infection is caused by single strain or by multiple strain and if recurrence of the disease is due to treatment failure or infection with new strain of *M. tuberculosis *[3, 4]. Spoligotyping, targeting the Direct Repeat locus and Mycobacterial Interspersed Repetitive Unit-Variable Number Tandem Repeats (MIRU-VNTRs) typing, has been shown to be a valuable alternative to IS*6110 *[5, 6]. An optimized 24-loci MIRU-VNTR typing scheme has been proposed as international standard [7, 8]. In addition to their use for tracing TB transmission at the strain level, MIRU-VNTR markers are also phylogenetically more informative, especially in the 24-locus format and can therefore be used to predict grouping into strain lineage [9, 10].

Although TB is still a public health problem in Iraq, there is little information about the genetic characteristics of the isolates driving the epidemic. A better knowledge of the molecular characteristics of *M. tuberculosis *complex isolates could contribute to better understanding of the transmission dynamics of the disease within the country and can guide interventions to control the epidemic. The aim of this study is to determine molecular epidemiology features of *M. tuberculosis* isolates in Baghdad, as well as in other Iraqi governorates, to assess their transmission dynamics.

## 2. Materials and Methods 

### 2.1. Study Population

This study was conducted in Baghdad at the Center of Chest and Respiratory diseases. A total of 270 isolates were collected between 2010 and 2011 representing approximately 40% of new and previously treated TB patients. This study was approved by the local ethical committee.

### 2.2. Culture and Drug Susceptibility Test

Diagnostic specimens were cultured and isolated on Lowenstein-Jensen (LJ) media after decontamination. Drug Susceptibility Tests (DST) against first-line anti-TB drugs rifampicin (RFP), isoniazid (INH), streptomycin (SM), and ethambutol (EMB) were combined with LJ medium at the following concentrations: RFP 40 *μ*g/mL, INH 0.2 *μ*g/mL, SM 4 *μ*g/mL, and EMB 2 *μ*g/mL. DST was used to detect the drug resistance of *M. tuberculosis* by the proportion method [11].

### 2.3. Identification of *Mycobacterium tuberculosis*

*Mycobacterium tuberculosis* isolates were identified on the basis of colony morphology, growth rate, pigmentation properties, niacin accumulation, nitrate reduction, thiophene-2-carboxylic acid hydrazide (TCH), and para-nitrobenzoic acid (PNB) test [11].

### 2.4. DNA Extraction

DNA was extracted from cultures by the standard Cetyltrimethyl ammonium-bromide (CTAB) method. The DNA was stored in TE buffer (10 mM Tris, 1 mM EDTA) at −20°C until use [12].

### 2.5. Spoligotyping

All genotyping methods were performed at the Emerging Bacterial Pathogens Unit, WHO/IUATLD Supra-National Reference TB Laboratory, San Raffaele Scientific Institute (FCSR). Spoligotyping analysis was performed by using commercial kit (Ocimun Biosolutions) as described by Kamerbeek et al. [13]. The 43 spacers between the direct repeats in the target region were amplified by using DRa biotinylated at 5′ end and DRb primers. The PCR products were hybridized to a membrane containing 43 oligonucleotides by reverse line blotting. *M. tuberculosis *H37Rv and* M. bovis* BCG were used as positive controls in each run. Spoligotyping results were converted into octal code and analyzed by using the SITVIT2 proprietary database of the Pasteur Institute of Guadeloupe, which is an updated version of the previously released SpolDB4 and SITVITWEB databases [14, 15].

### 2.6. MIRU-VNTR

Standardized 24-loci MIRU-VNTR typing [7] was performed using the MIRU-VNTR typing kit (Genoscreen, Lille, France). PCR products were run with Genescan 1200LIZ size standard (Applied Biosystems, California, and USA) on ABI3730 sequencer. Sizing of the PCR fragments and assignment of MIRU-VNTR alleles were done by Gene Mapper software version 3.7 (Applied Biosystems, California, USA). In order to deine clusters and to build an UPMGA tree, we used the MIRU-VNTRplus web application available at http://www.miru-vntrplus.org/MIRU/index.faces. The allelic diversity of the strains was determined by using the Hunter Gaston Discriminatory Index (HGDI).

### 2.7. Interpretation of Typing Results 

Spoligotypes in binary format and MIRU patterns in 24-digit codes were entered in the SITVIT2 database. At the time of the comparison, it contained genotyping data on more than 100,000 MTBC strains isolated from 160 countries of patient origin. In this database, “SIT” (Spoligotype International Type) designates spoligotyping shared by two or more patient isolates, “MIT” (MIRU International Type) designates 24-loci MIRU patterns shared by two or more patient isolates, and “orphan” designates patterns reported for a single isolate. Major spoligotyping-based phylogenetic clades were assigned according to revised signatures provided in SITVITWEB [15]. These clades include specific signatures for *M. tuberculosis *complex members and rules defining major lineages/sublineages for *M. tuberculosis *sensu stricto, that is, the Central Asian (CAS) clade and 2 sublineages, the East-African-Indian (EAI) clade and 9 sublineages, the Haarlem (H) clade and 3 sublineages, the Latin-American-Mediterranean (LAM) clade and 12 sublineages note that two LAM sublineages were recently raised to lineage level: LAM10-CAM as the Cameroon lineage [16] and LAM7-TUR as the Turkey lineage [17], the “Manu” family and 3 sublineages, the IS*6110*-low-banding X clade and 4 sublineages, and an ill-defined T clade with 5 sublineages. The recently described “Ural” family, subdivided into 2 sublineages (Ural-1 and Ural-2), replaced some spoligotype patterns previously classified as H3 and H4 [18].

### 2.8. Phylogenetic Analysis

The evolutionary relationships among all the observed spoligotypes were studied by drawing minimum spanning trees (MSTs) with BioNumerics Software version 6.6 (Applied Maths, Sint-Martens-Latem, Belgium).

## 3. Results

### 3.1. Studied Population

This study was performed over a period of 13 months on 270 *M. tuberculosis *complex strains from Iraqi patients. The related demographic information and drug resistance patterns obtained are summarized in Table 1. One hundred and fifty-seven (58.14%) of the cases included in the study were from Baghdad, 113 (41.85%) were from other governorates in Iraq but diagnosed in Baghdad. As shown in the table, male patients are predominants (male to female sex ratios varied from 1.49 to 3.04 depending on the origin of the patients, from Baghdad or other governorates, resp.) and 56.7% of the TB patients included in the study are between 21 and 40 years old with the mean age 36.3. As for smear microscopy, 167 tested positive and 103 were negative. 187 patients had newly TB diagnosed, while 83 patients were previously treated cases (PTC). Lastly, 171 cases were susceptible to all antituberculosis drugs, whereas 64/270 (23.7%) patients had MDR. 35 patients had an unknown resistance profile.

### 3.2. Distribution of Phylogenetic Clades

Spoligotyping results are summarized in Tables 2 and 3. A total of 117 different patterns were observed among 270 strains studied; a total of 53 patterns corresponding to 53 strains belonged to orphan patterns not yet reported to the SITVIT2 database (Table 2), whereas the remaining 64 patterns (*n* = 217) corresponded to SITs, of which 48 patterns (*n* = 173 strains) matched a preexisting shared type in the SITVIT2 database, while 16 SITs (*n* = 44 strains) were newly created. Of these, 6 SITs (SIT3346, SIT3497, SIT3708, SIT3790, SIT3791, and SIT3800) (*n* = 32 strains) were created within the present study and 10 were created after a match with an orphan in the database (SIT3275 from France metropolitan area, SIT3789 from Iraq, SIT3792, SIT3793, and SIT3794 from India, SIT3795 and SIT3799 from Brazil, SIT3796 from Pakistan, SIT3797 from Mexico, and SIT3798 from Iraq).

**Figure 1:**
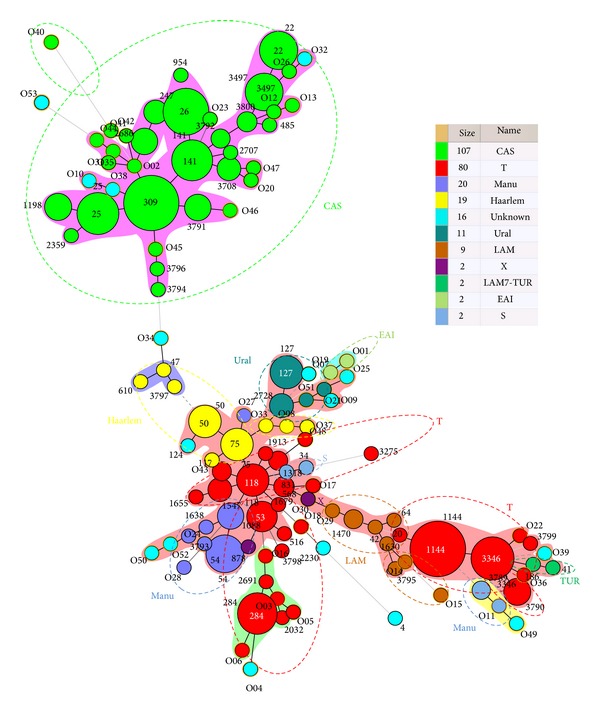
A minimum spanning tree (MST) illustrating evolutionary relationships between the *Mycobacterium tuberculosis* spoligotypes. This kind of tree connects each genotype based on degree of changes required to go from one allele to another. The structure of the tree is represented by branches (continuous versus dashed and dotted lines) and circles representing each individual pattern. Note that the length of the branches represents the distance between patterns while the complexity of the lines (continuous, gray dashed, and gray dotted) denotes the number of allele/spacer changes between two patterns: solid lines represent 1, 2, or 3 changes (thicker ones indicate a single change, while the thinner ones indicate 2 or 3 changes); gray dashed lines represent 4 changes; and gray dotted lines represent 5 or more changes. The size of the circle is proportional to the total number of isolates in our study, illustrating unique isolates (smaller nodes) versus clustered isolates (bigger nodes). The color of the circles indicates the phylogenetic lineage to which the specific pattern belongs. Note that orphan patterns are circled in orange. Patterns colored in cyan-blue indicate a strain with an unknown signature (unclassified).

Nearly 94.1% of the isolates belonged to known genotype clades. These include, in decreasing order: CAS 39.6%, ill-defined T clade 29.6%, Manu 7.4%, Haarlem 7%, Ural 4.1%, LAM 3.3%, X 0.7%, LAM7-TUR 0.7%, EAI 0.7%, S 0.7%, and unknown 5.9%. This observation on the complex diversity of *M. tuberculosis* in Iraq was further supported by the minimum spanning tree (MST) illustrated in Figure 1, which was constructed on all isolates (*n* = 270, including 53 orphan patterns).

As shown in Figure 1, the CAS lineage strains constitute the biggest group of strains infecting Iraqi patients; the major shared types being SIT309, 26, 141, 25, 3497, and 22. At a considerable distance is seen a separate group of strains made up of smaller nodes: T lineage (SIT1144, 3346, 284, 118, 53), Haarlem (SIT75, 50), LAM (SIT42, 1470), Ural (SIT127), and Manu (SIT54). Even smaller nodes concerned other lineages such as Turkey, S, X, and the ancestral EAI lineages. Indeed, as many as 129/270 (47.7%) strains from this study belonged to Principal Genetic Group 1 (PGG1), according to the *KatG-gyrA* polymorphism-based classification of Sreevatsan et al. [19], and these strains represent all of known lineages associated with PGG1 strains (CAS, Manu, and EAI with the exception of Beijing). However, evolutionary modern PGG2/3 strains constitute a high proportion in this study such as ill-defined T, Haarlem, Ural, LAM, LAM7-TUR, S, and X. These strains accounted for 125/270 (46.3%) of the studied strains.

A total of 31/64 SITs containing 184 strains were clustered within this study (2 to 17 strains per cluster) while 33/64 SITs containing 33 strains were unique. For total unique strains, one should add to this number the 53 orphan strains, which brings the number of unclustered strains in this study to 86/270 or 31.85% and clustered strains to 184/270 or 68.15%. 23 clusters are shown in Table 3, along with 8 additional clusters (2 to 11 strains per cluster) represented by the newly created shared types.

The two largest clusters of 17 strains were composed of SIT309 (CAS1-Delhi lineage) and SIT1144 (T1 lineage).

Description of clusters containing 5 or more isolates in this study, and their worldwide distribution in the SITVIT2 database, is detailed in Table 4 and will be commented further under the discussion section.

**Figure 2:**
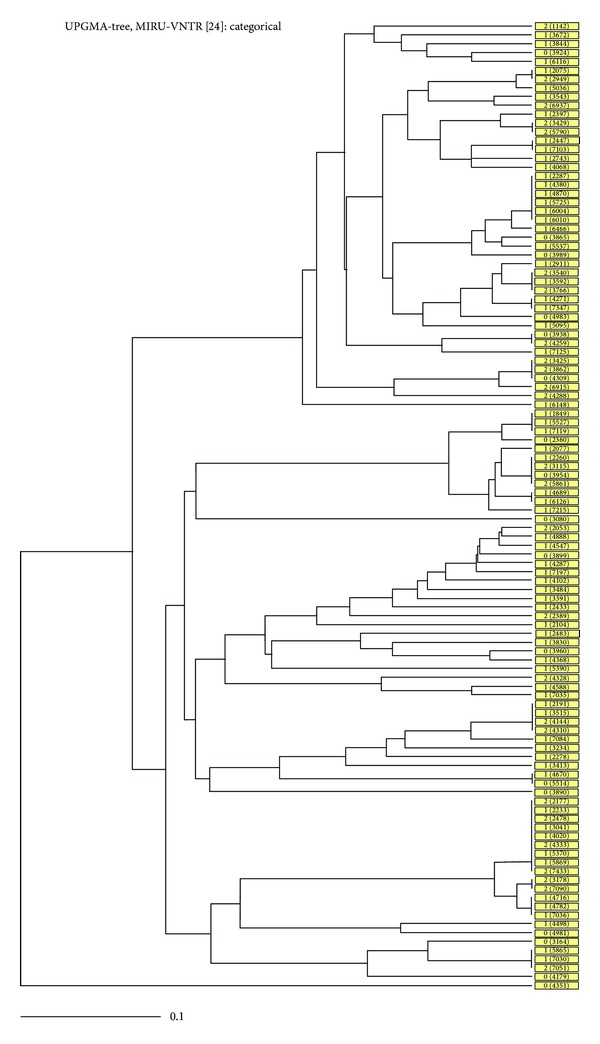
UPGMA tree based on MIRU 24 pattern of the subset of 110 samples. Drug resistance (Drug R) information is shown as 0, unknown; 1, non-MDR; 2, MDR-TB, that is, combined resistance to INH-RIF (with or without resistance to other drugs).

The MIRU-VNTR analysis detected a total of 73 MIRU patterns from 110 strains using the full 24 MIRU-VNTR locus set, including 17 clusters and 56 unique (Figure 2). Allelic diversity for each locus was calculated in order to determine the discriminatory power of these loci in a combined group for the *Mycobacterium tuberculosis* population studied. Based on their discriminatory index (HGDI), 7 loci (MIRU02, MIRU04, MIRU20, Mtub29, ETR-B, MIRU24, and MIRU27) showed poor discriminatory power (HGDI < 0.3). Seven loci (MIRU 23, Qub11b, Mtub30, Mtub34, Mtub39, MIRU 39, and QUB 4156) discriminated the isolated moderately (0.3 ≤ HGDI ≤ 0.6). Lastly, 10 loci (Mtub 04, ETR-C, MIRU 40, MIRU 10, MIRU 16, Mtub21, ETR-A, MIRU 26, MIRU 31, and QUB-26) were highly discriminative (HGDI > 0.6). In this study the locus QUB-26 was found to be the most discriminatory allele in order to distinguish between strains (HGDI of 0.83). Conversely, locus MIRU-24 was found to be the least discriminatory with an HGDI of 0.03. The Recent Transmission Index (RTI_*n*−1_) was found at 33.6% showing evidence of ongoing transmission.

### 3.3. Drug Resistance Patterns

**Figure 3:**
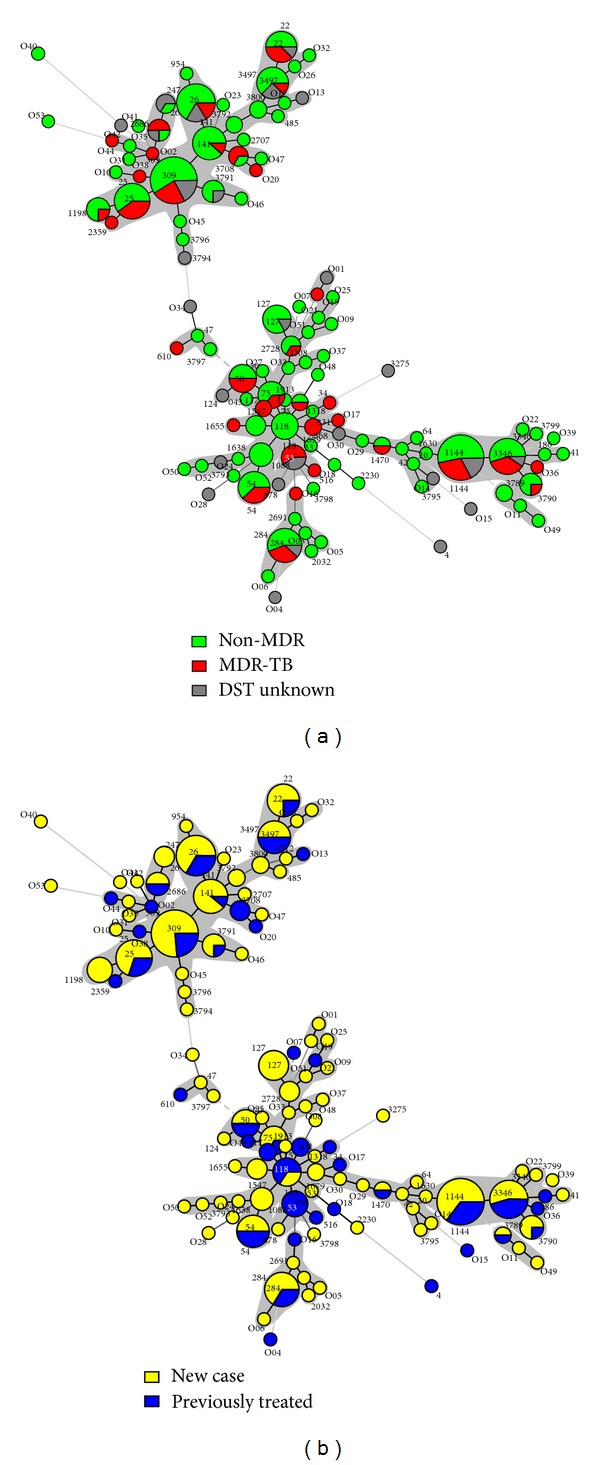
A minimum spanning tree (MST) illustrating evolutionary relationships between the *M. tuberculosis* spoligotypes in our study in function to studied parameters. (a) Drug resistance and (b) treatment status, that is, new versus retreated cases.

Both the drug resistance patterns and the treatment status of the patients (new versus retreated cases) were studied in detail on all the 270 strains included in this study in function of their spoligotyping-based genotypic lineages, and the results were concomitantly exploited to draw a minimum spanning tree (MST) shown in Figure 3. Of the 270 strains studied, 171 (63.3%) were sensitive to all five of the first-line drugs tested, 64 (23.70%) were MDR, while the drug-susceptibility information was not available for 35 (12.96%) of strains. Regarding the treatment status of the patients, 187 (69.26%) were new cases while 83 (30.74%) were previously treated. It is noteworthy that all the 64 MDR cases were exclusively found among the retreated patients, bringing the proportion of MDR isolates in this group to 77.1% the difference of drug resistance between new versus retreated cases being highly significant (*P* value < 0.0001). As shown in Figure 3(a), a high frequency of MDR-TB was associated with SIT53/T1 which contained 60% of MDR strains. Interestingly, this same shared type was also exclusively associated with retreated cases (Figure 3(b)). Although the clustering rate between drug susceptible and drug resistant isolates did not vary significantly (*P* value > 0.4), differences were noted when comparing predominant SITs. Indeed, one may notice a relatively high frequency of MDR-TB among patterns related to SIT53/T1 (60% of MDR) in Figure 3(a), which was significantly higher than other SITs such as SIT26/CAS1-Delhi (16.67% of MDR), *P* value = 0.0372, and SIT141/CAS1-Delhi (11.11% of MDR), *P* value = 0.0030. Lastly, although the rate of MDR-TB was slightly higher among patients from cities other than Baghdad (Table 1), the difference was not statistically significant (*P* value = 0.1224).

## 4. Discussion

In this study we characterized, by spoligotyping, 270 *M. tuberculosis* isolates collected from patients diagnosed in Baghdad/Reference TB laboratory in a 13-month period. One hundred and ten samples were also characterized by 24-loci MIRU-VNTR.

All TB cases reported in this study were caused by *M. tuberculosis*. The molecular investigation of strain by spoligotyping did not show the specific indicator for other members of *M. tuberculosis *complex such as *M. bovis*. This situation has been described in other settings and countries such as by Godreuil et al. in west African countries [20], Nakajima et al. in Bangladesh [21], and Viegas in Mozambique [22]. Spoligotyping of the 270 *M. tuberculosis* strains revealed 31 clusters consisting of 184 strains, with clustering rate 57%, whereas 86 strains were unique.

One hundred and seven (39.6%) of the 270 studied strains were CAS belonging to different SITs. CAS has also been identified as predominant family in Saudi Arabia (22.5%) and also the predominance of Delhi genogroup in Iran [23, 24] was reported. In Pakistan this lineage represents 61% of the total [25]. And CAS has been also identified by recent study as a predominant strain in north India [26]. The data from Turkey suggested that there is no dominant *M. tuberculosis* clade such as what has been observed in Asia and former USSR republics [17]. CAS clade is followed, in decreasing order, by ill-defined T clade 29.6%, Manu 7.4%, Haarlem 7%, Ural 4.1%, LAM 3.3%, X 0.7%, LAM7-TUR 0.7%, EAI 0.7%, S 0.7%, and unknown 5.9% (Table 4). These observations emphasize the complex diversity of circulating *M. tuberculosis* strains in Iraq that could reflect the different transmission pathways occurring within the country. Besides, it has been suggested that particular lineages of *M. tuberculosis* might be adapted to specific human population [27, 28]. Indeed, many strains of this study belonged to Principal Genetic Group (PGG1), according to the *KatG-grA* polymorphism-based classification of Sreevatsan et al. [19], and these strains represent all of the known lineages associated with PGG1 with the exception of the Beijing clade. However, evolutionary modern PGG 2/3 strains were also found with different distribution.

In this study 110 strains were classified into 73 MIRU patterns of which 17 were clusters and 56 unique strains. The high level of Recent Transmission Index (RTI), at 33.6%, indicates that the most cases are due to recent transmissions rather than reactivation of *M. tuberculosis* infections. In our study, ten loci (Mtub 04, ETR-C, MURU 40, MIRU 10, MIRU 16, Mtub21, ETR-A, MIRU 26, MIRU 31, and QUB-26) were highly discriminatory (HGDI > 0.6), seven loci (MIRU23, Qub11b, Mtub30, Mtub34, Mtub39, MIRU 39, and QUB 4156) moderately discriminate (0.3 ≤ HGDI ≤ 0.6), and seven loci (MIRU02, MIRU04, MIRU20, Mtub 29, ETR-B, MIRU 24, and MIRU27) showed poor discriminatory power (HGDI < 0.3).

MIRU-VNTR allelic results have been correlated with definition of ancestral and modern MTB lineages, with the presence of single allele in locus 24 being related to a modern strain type. We found that 98% of our strains contained only single repeat at locus 24, further confirming their modern lineage. This is comparable with previous reports for CAS strain from Bangladesh and Singapore [29, 30] and also with the studies from Pakistan and Bulgaria as supported by the finding that 62% of their CAS family strains contained only one allele at the locus 24 [31, 32]. Moreover, the relative discriminatory powers of particular VNTR loci vary depending on the strain in question [33–37].

This study found that in Iraqi population, the characteristic of MDR in *M. tuberculosis* is mostly acquired as a result of treatment failure, due to irregularity in taking of drug (anti-TB), neglect, and incorrect prescriptions. Although the extremely high level of MDR among previously treated patients might indicate transmission chains within the population, molecular epidemiology revealed that except for SIT53/T1 genotype, no significant differences were found and the MIRU analysis in the subset of strains did not show clusters of exclusively MDR strains. The fact that identical MIRU patterns were shared both by MDR and non-MDR strains, and that the isoniazid and rifampicin resistance patterns were independent of their genotypes, suggests that MDR strains most probably emerged due to the selective pressure because of problems in adherence to treatment (in addition to other environmental factors), (high population, poor housing, overcrowding, and malnutrition).

The data of this study provide important baseline information on the genetic diversity of *M. tuberculosis* in Iraq. Therefore, it could be used to monitor change in the transmission pattern of tuberculosis. Spoligotyping has been proved useful for categorizing strains into different families and can be used as an initial technique to be subsequently followed by MIRU-VNTR. This study showed that 24-loci MIRU-VNTR typing offers a higher discriminatory power. Iraq needs to conduct epidemiological survey by using conventional and genotyping methods in order to provide adequate data that can be used for the formulation of control strategies of tuberculosis transmission.

## Figures and Tables

**Table 1:** Demographic characteristics of the population studied and drug resistance profiles.

Parameters	Origin of patients		
	Baghdad (%)	Other governorates (%)	Total
Total no. of strain	157 (58.1)	113 (41.9)	270
Sex			
Male	94 ( 59.9)	85 (75.2)	179
Female	63 (40.1)	28 (24.8)	91
Sex ratio M : F	1.49	3.04	1.97
Age group (yr) (no.)			
0–20	17 (10.8)	4 (3.5)	21
21–40	82 (52.2)	71 (62.8)	153
41–60	48 (30.6)	25 (22.1)	73
>60	10 (6.4)	13 (11.5)	23
Smear microscopy (no.)			
Positive	98 (62.4)	69 (61.1)	167 (61.8)
Negative	59 (37.6)	44 (38.9)	103 (38.2)
Treatment			
New cases	119 (75.8)	68 (60.2)	187 (69.3)
P.T.C	38 ( 24.2)	45 (39.8)	83 (30.7)
Drug susceptibility			
Susceptible to all drugs	102 (65)	69 (61.1)	171 (63.3)
MDR (INH − RIF)	31 (19.8)	33 (29.2)	64 (23.7)
Unknown	24 (15.3)	11 (9.7)	35 (13.0)

**Table 2:** Orphan strains (*n* = 53) and corresponding spoligotyping defined lineages/sublineages recorded among a total of 270 *M. tuberculosis* strains (one isolate per patient) from Iraqi patients.

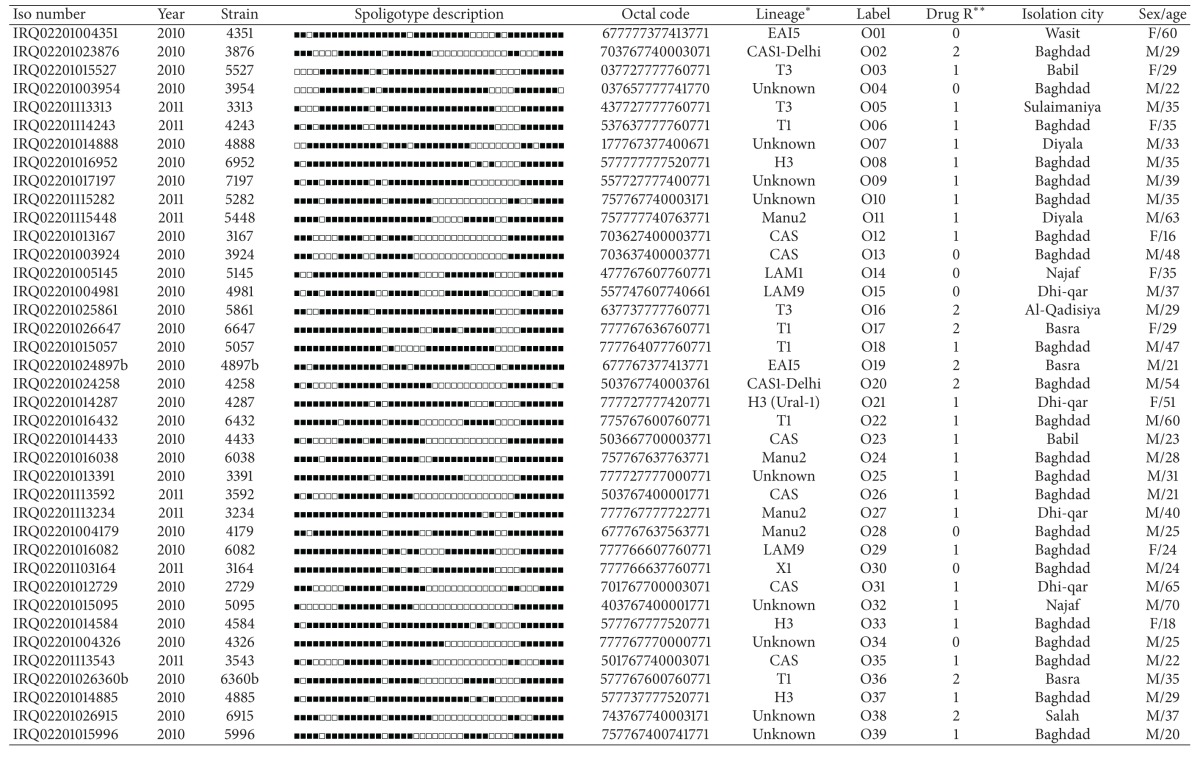 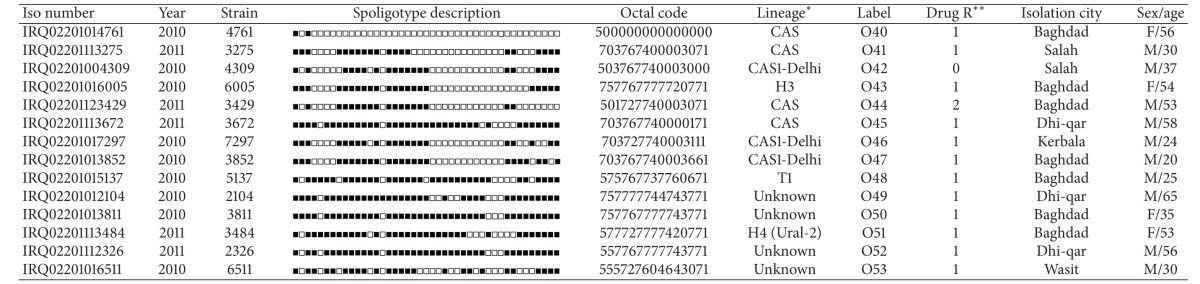

*Lineage designations for orphan patterns were done manually as expert-based interpretations using revised SpolDB4 rules.

**Drug resistance (drug R) information is shown as 0, unknown; 1, non-MDR; 2, MDRTB, that is, combined resistance to INH − RIF (with or without resistance to other drugs); 3, any other resistance(s); 4, proven XDRTB, that is, resistance to INH + RIF + a fluoroquinolone + any 1 of 3 injectable 2nd-line drugs (capreomycin, kanamycin, amikacin).

**Table 3:** Description of 64 shared types (SITs; *n* = 217 isolates) and corresponding spoligotyping defined lineages/sublineages starting from a total of 270 *M. tuberculosis* strains isolated from Iraqi patients.

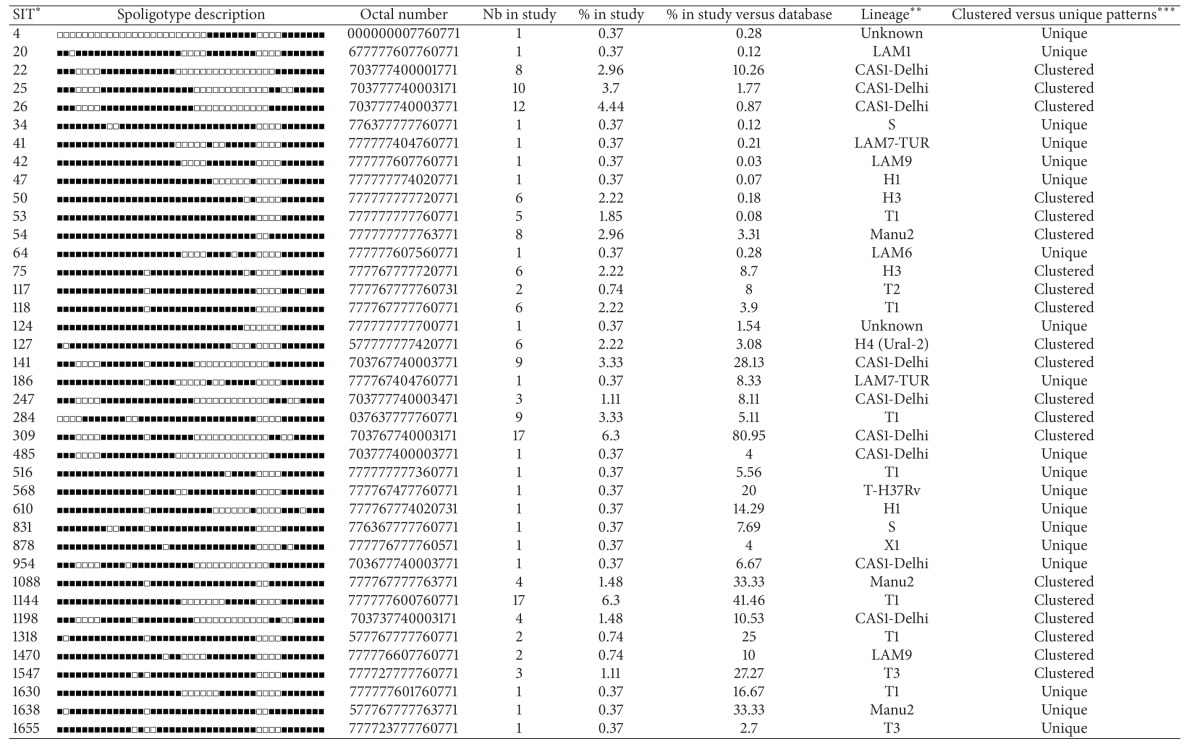 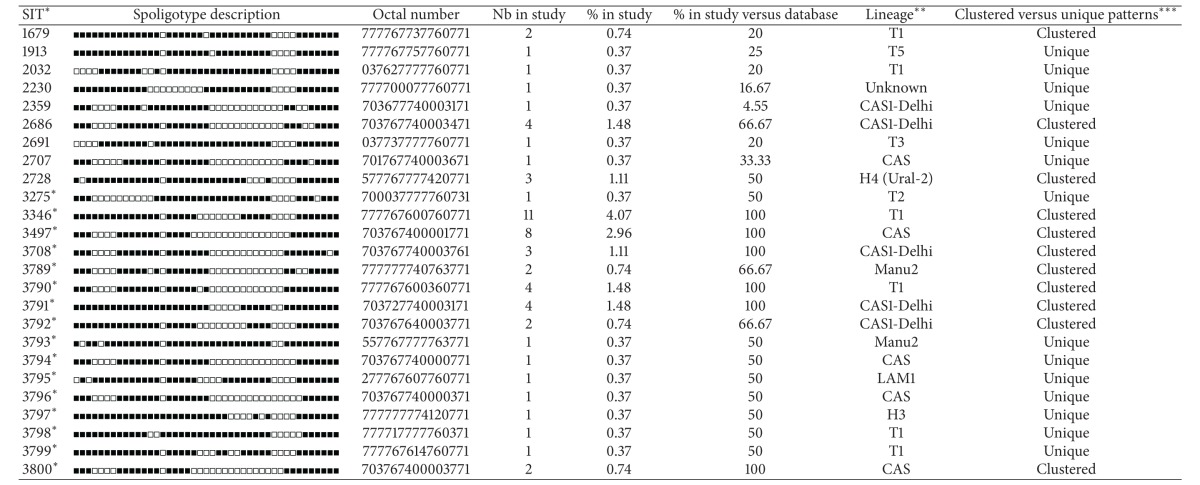

*A total of 48/64 SITs containing 173 isolates matched a preexisting shared type in the database, whereas 16/64 SITs (*n* = 44 isolates) were newly created. A total of 31/64 SITs containing 184 isolates were clustered within this study (2 to 17 isolates per cluster) while 33/64 SITs containing 33 strains were unique (for total unique strains, one should add to this number the 53 orphan strains, which brings the number of unclustered isolates in this study to 86/270 or 31.85% and clustered isolates to 184/270 or 68.15%). Note that SITs followed by an asterisk indicates “newly created” SITs due to 2 or more strains belonging to an identical new pattern within this study or after a match with an orphan in the database; SIT designations followed by number of strains: 3275* this study *n* = 1, FXX *n* = 1; 3346* this study *n* = 11; 3497* this study *n* = 8; 3708* this study *n* = 3; 3789* this study *n* = 2, IRQ *n* = 1; 3790* this study *n* = 4; 3791* this study *n* = 4; 3792* this study *n* = 2, IND *n* = 1; 3793* this study *n* = 1, IND *n* = 1; 3794* this study *n* = 1, IND *n* = 1; 3795* this study *n* = 1, BRA *n* = 1; 3796* this study *n* = 1, PAK *n* = 1; 3797* this study *n* = 1, MEX *n* = 1; 3798* this study *n* = 1, IRQ *n* = 1; 3799* this study *n* = 1, BRA *n* = 1; 3800* this study *n* = 2.

**Lineage designations according to SITVIT2 using revised SpolDB4 rules; “unknown” designates patterns with signatures that do not belong to any of the major lineages described in the database.

***Clustered strains correspond to a similar spoligotype pattern shared by 2 or more strains “within this study,” as opposed to unique strains harboring a spoligotype pattern that does not match with another strain from this study. Unique strains matching a preexisting pattern in the SITVIT2 database are classified as SITs, whereas in case of no match, they are designated as “orphan” (see Table 2).

**Table 4:** Description of clusters containing 5 or more isolates in this study and their worldwide distribution in the SITVIT2 database.

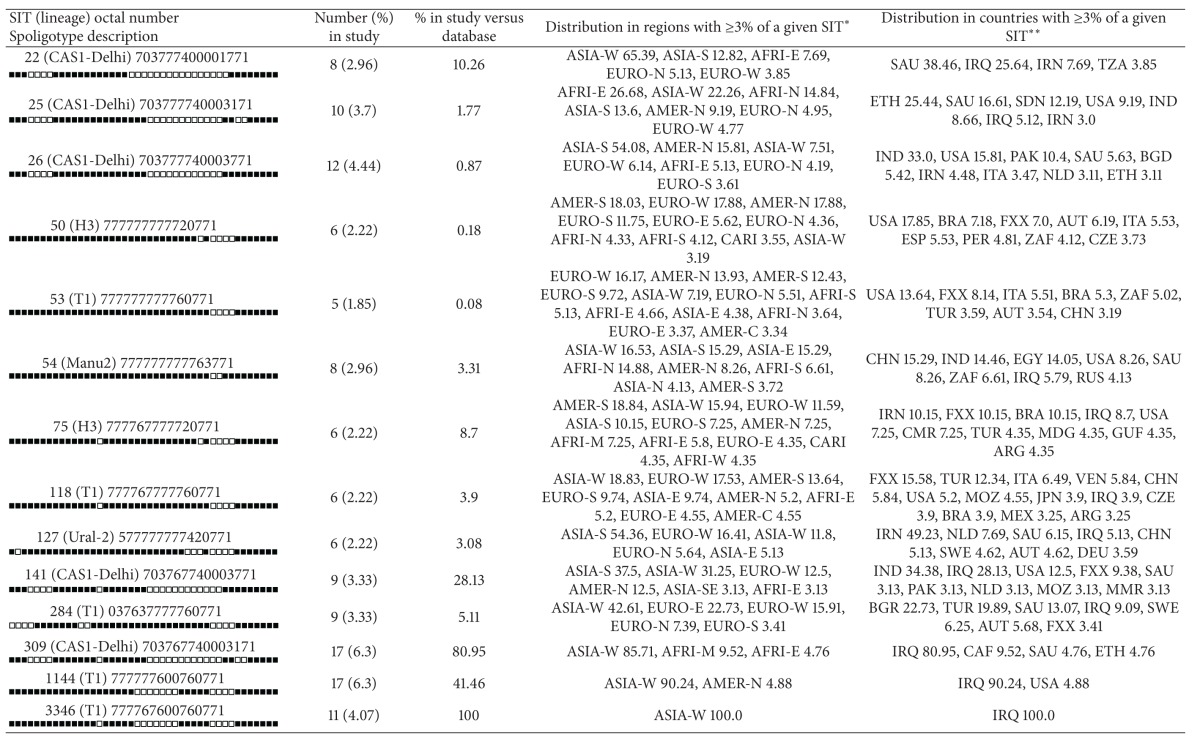 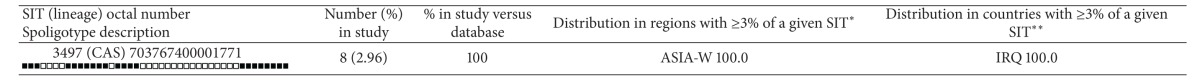

*Worldwide distribution is reported for regions with more than 3% of a given SITs as compared to their total number in the SITVIT2 database. The definition of macrogeographical regions and subregions (http://unstats.un.org/unsd/methods/m49/m49regin.htm) is according to the United Nations; regions: AFRI (Africa), AMER (Americas), ASIA (Asia), EURO (Europe), and OCE (Oceania) are subdivided in E (eastern), M (middle), C (central), N (northern), S (southern), SE (south eastern), and W (western). Furthermore, CARIB (caribbean) belongs to Americas, while Oceania is subdivided in 4 subregions, AUST (Australasia), MEL (Melanesia), MIC (Micronesia), and POLY (Polynesia). Note that in our classification scheme, Russia has been attributed a new subregion by itself (northern Asia) instead of including it among the rest of eastern Europe. It reflects its geographical localization as well as due to the similarity of specific TB genotypes circulating in Russia (a majority of Beijing genotypes) with those prevalent in central, eastern, and south eastern Asia.

**The 3 letter country codes are according to http://en.wikipedia.org/wiki/ISO_3166-1_alpha-3; countrywide distribution is only shown for SITs with ≥3% of a given SITs as compared to their total number in the SITVIT2 database.

## References

[1] WHO (2012). Global tuberculosis control. *WHO Report*.

[2] Ministry of Health of Iraq (2012). New diagnosis of tuberculosis in Iraq.

[3] Mathema B, Kurepina NE, Bifani PJ, Kreiswirth BN (2006). Molecular epidemiology of tuberculosis: current insights. *Clinical Microbiology Reviews*.

[4] Kontsevaya IS, Nikolayevsky VV, Balabanova YM (2011). Molecular epidemiology of tuberculosis: objectives, methods, and prospects. *Molecular Genetics, Microbiology and Virology*.

[5] Supply P, Lesjean S, Savine E, Kremer K, van Soolingen D, Locht C (2001). Automated high-throughput genotyping for study of global epidemiology of *Mycobacterium tuberculosis* based on mycobacterial interspersed repetitive units. *Journal of Clinical Microbiology*.

[6] Cowan LS, Diem L, Monson T (2005). Evaluation of a two-step approach for large-scale, prospective genotyping of *Mycobacterium tuberculosis* isolates in the United States. *Journal of Clinical Microbiology*.

[7] Supply P, Allix C, Lesjean S (2006). Proposal for standardization of optimized mycobacterial interspersed repetitive unit-variable-number tandem repeat typing of *Mycobacterium tuberculosis*. *Journal of Clinical Microbiology*.

[8] Thong-On A, Smittipat N, Juthayothin T (2010). Variable-number tandem repeats typing of *Mycobacterium tuberculosis* isolates with low copy numbers of IS6110 in Thailand. *Tuberculosis*.

[9] Wirth T, Hildebrand F, Allix-Béguec C (2008). Origin, spread and demography of the *Mycobacterium tuberculosis* complex. *PLoS Pathogens*.

[10] Cardoso Oelemann M, Gomes HM, Willery E (2011). The forest behind the tree: phylogenetic exploration of a dominant *Mycobacterium tuberculosis* strain lineage from a high tuberculosis burden country. *PloS ONE*.

[11] David H, Levy-Frébault V, Thorel MF (1989). *Méthodes de laboratoire de mycobactériologie clinique*.

[12] van Soolingen D, Hermans PWM, de Haas PEW, Soll DR, van Embden JDA (1991). Occurrence and stability of insertion sequences in *Mycobacterium tuberculosis* complex strains: evaluation of an insertion sequence-dependent DNA polymorphism as a tool in the epidemiology of tuberculosis. *Journal of Clinical Microbiology*.

[13] Kamerbeek J, Schouls L, Kolk A (1997). Simultaneous detection and strain differentiation of *Mycobacterium tuberculosis* for diagnosis and epidemiology. *Journal of Clinical Microbiology*.

[14] Brudey K, Driscoll JR, Rigouts L (2006). *Mycobacterium tuberculosis* complex genetic diversity: mining the fourth international spoligotyping database (SpolDB4) for classification, population genetics and epidemiology. *BMC Microbiology*.

[15] Demay C, Liens B, Burguière T (2012). SITVITWEB—a publicly available international multimarker database for studying *Mycobacterium tuberculosis* genetic diversity and molecular epidemiology. *Infection, Genetics and Evolution*.

[16] Koro Koro F, Simo YK, Piam FF (2013). Population dynamics of tuberculous Bacilli in Cameroon as assessed by spoligotyping. *Journal of Clinical Microbiology*.

[17] Kisa O, Tarhan G, Gunal S (2012). Distribution of spoligotyping defined genotypic lineages among drug-resistant *Mycobacterium tuberculosis* complex clinical isolates in Ankara, Turkey. *PLoS ONE*.

[18] Mokrousov I (2012). The quiet and controversial: ural family of *Mycobacterium tuberculosis*. *Infection, Genetics and Evolution*.

[19] Sreevatsan S, Pan X, Stockbauer KE (1997). Restricted structural gene polymorphism in the *Mycobacterium tuberculosis* complex indicates evolutionarily recent global dissemination. *Proceedings of the National Academy of Sciences of the United States of America*.

[20] Godreuil S, Torrea G, Terru D (2007). First molecular epidemiology study of *Mycobacterium tuberculosis* in Burkina Faso. *Journal of Clinical Microbiology*.

[21] Nakajima C, Rahim Z, Fukushima Y (2010). Identification of *Mycobacterium tuberculosis* clinical isolates in Bangladesh by a species distinguishable multiplex PCR. *BMC Infectious Diseases*.

[22] Viegas SO, Machado A, Groenheit R (2010). Molecular diversity of *Mycobacterium tuberculosis* isolates from patients with pulmonary tuberculosis in Mozambique. *BMC Microbiology*.

[23] Al-Hajoj SA (2010). Tuberculosis in Saudi Arabia: can we change the way we deal with the disease?. *Journal of Infection and Public Health*.

[24] Jafarian M, Aghali-Merza M, Farnia P, Ahmadi M, Masjedi MR, Velayati AA (2010). Synchronous comparison of *Mycobacterium tuberculosis* epidemiology strains by MIRU-VNTR and MIRU-VNTR and spoligotyping technique. *Avicenna Journal of Medical Biotechnology*.

[25] Tanveer M, Hasan Z, Siddiqui AR (2008). Genotyping and drug resistance patterns of *M. tuberculosis* strains in Pakistan. *BMC Infectious Diseases*.

[26] Varma-Basil M, Kumar S, Arora J (2011). Comparison of spoligotyping, mycobacterial interspersed repetitive units typing and IS6110-RFLP in a study of genotypic diversity of *Mycobacterium tuberculosis* in Delhi, North India. *Memórias do Instituto Oswaldo Cruz Rio de Janeiro*.

[27] Helal ZH, Ashour MSE-D, Eissa SA (2009). Unexpectedly high proportion of ancestral manu genotype *Mycobacterium tuberculosis* strains cultured from tuberculosis patients in Egypt. *Journal of Clinical Microbiology*.

[28] Gagneux S, DeRiemer K, Van T (2006). Variable host-pathogen compatibility in *Mycobacterium tuberculosis*. *Proceedings of the National Academy of Sciences of the United States of America*.

[29] Banu S, Gordon SV, Palmer S (2004). Genotypic analysis of *Mycobacterium tuberculosis* in bangladesh and prevalence of the Beijing strain. *Journal of Clinical Microbiology*.

[30] Sun Y-J, Bellamy R, Lee ASG (2004). Use of mycobacterial interspersed repetitive unit-variable-number tandem repeat typing to examine genetic diversity of *Mycobacterium tuberculosis* in Singapore. *Journal of Clinical Microbiology*.

[31] Ali A, Hasan Z, Tanveer M (2007). Characterization of *Mycobacterium tuberculosis* central Asian Strain 1 using mycobacterial interspersed repetitive unit genotyping. *BMC Microbiology*.

[32] Valcheva V, Mokrousov I, Narvskaya O, Rastogi N, Markova N (2008). Utility of new 24-locus variable-number tandem-repeat typing for discriminating *Mycobacterium tuberculosis* clinical isolates collected in Bulgaria. *Journal of Clinical Microbiology*.

[33] Murase Y, Mitarai S, Sugawara I, Kato S, Maeda S (2008). Promising loci of variable numbers of tandem repeats for typing Beijing family *Mycobacterium tuberculosis*. *Journal of Medical Microbiology*.

[34] Comas I, Homolka S, Niemann S, Gagneux S (2009). Genotyping of genetically monomorphic bacteria: DNA sequencing in *Mycobacterium tuberculosis* highlights the limitations of current methodologies. *PLoS ONE*.

[35] Ferdinand S, Valétudie G, Sola C, Rastogi N (2004). Data mining of *Mycobacterium tuberculosis* complex genotyping results using mycobacterial interspersed repetitive units validates the clonal structure of spoligotyping-defined families. *Research in Microbiology*.

[36] Aminian M, Shabbeer A, Bennett KP (2010). A conformal Bayesian network for classification of *Mycobacterium tuberculosis* complex lineages. *BMC Bioinformatics*.

[37] Mendes NH, Melo FAF, Santos ACB (2011). Characterization of the genetic diversity of *Mycobacterium tuberculosis* in São Paulo city, Brazil. *BMC Research Notes*.

